# Nipah Virus Transmission in a Hamster Model

**DOI:** 10.1371/journal.pntd.0001432

**Published:** 2011-12-13

**Authors:** Emmie de Wit, Trenton Bushmaker, Dana Scott, Heinz Feldmann, Vincent J. Munster

**Affiliations:** 1 Laboratory of Virology, Division of Intramural Research, National Institute of Allergy and Infectious Diseases, National Institutes of Health, Hamilton, Montana, United States of America; 2 Rocky Mountain Veterinary Branch, Division of Intramural Research, National Institute of Allergy and Infectious Diseases, National Institutes of Health, Hamilton, Montana, United States of America; 3 Department of Medical Microbiology, University of Manitoba, Winnipeg, Manitoba, Canada; University of Texas Medical Branch at Galveston, United States of America

## Abstract

Based on epidemiological data, it is believed that human-to-human transmission plays an important role in Nipah virus outbreaks. No experimental data are currently available on the potential routes of human-to-human transmission of Nipah virus. In a first dose-finding experiment in Syrian hamsters, it was shown that Nipah virus was predominantly shed via the respiratory tract within nasal and oropharyngeal secretions. Although Nipah viral RNA was detected in urogenital and rectal swabs, no infectious virus was recovered from these samples, suggesting no viable virus was shed via these routes. In addition, hamsters inoculated with high doses shed significantly higher amounts of viable Nipah virus particles in comparison with hamsters infected with lower inoculum doses. Using the highest inoculum dose, three potential routes of Nipah virus transmission were investigated in the hamster model: transmission via fomites, transmission via direct contact and transmission via aerosols. It was demonstrated that Nipah virus is transmitted efficiently via direct contact and inefficiently via fomites, but not via aerosols. These findings are in line with epidemiological data which suggest that direct contact with nasal and oropharyngeal secretions of Nipah virus infected individuals resulted in greater risk of Nipah virus infection. The data provide new and much-needed insights into the modes and efficiency of Nipah virus transmission and have important public health implications with regards to the risk assessment and management of future Nipah virus outbreaks.

## Author Summary

Understanding how viruses are transmitted plays an important role in our ability to intervene in virus outbreaks. Over the last decade, Nipah virus has caused multiple outbreaks in Malaysia, India and especially Bangladesh. Fruit bats form the natural reservoir for Nipah virus; from the bats the virus is introduced into the human population, either directly or via an intermediate host. Epidemiological data suggest that upon introduction into the human population the virus has the ability to spread from person-to-person. We performed experimental studies in a hamster model to investigate if we could mimic human-to-human transmission and to determine the route of transmission through which Nipah virus spread between people. We discovered that Nipah virus-infected hamsters predominantly shed virus via excretions from the nose and lungs. In transmission experiments, we showed that Nipah virus is efficiently transmitted via direct contact. Fomite transmission was inefficient and transmission via aerosols did not occur. The elucidation of the mode of Nipah virus transmission has important public health implications because it allows a targeted and experiment-based assessment of intervention strategies and surveillance for emerging Nipah virus strains better adapted to human-to-human transmission.

## Introduction

Nipah virus is a member of the *Henipavirus* genus in the *Paramyxoviridae* family. Nipah virus first emerged in humans in Malaysia in 1998–1999, during a large outbreak of encephalitis and respiratory disease in humans, causing 276 cases of encephalitis, with 106 fatalities [1]. The subsequent detection of antibodies against Nipah virus, Nipah viral RNA and the isolation of Nipah virus from samples of Pteropus spp fruit bats indicated that they form the natural reservoir of Nipah virus [2], [3], [4], [5]. In Malaysia, Nipah virus-infected pigs formed the intermediate and amplifying host in the transmission cycle from the natural reservoir to humans [6]. The second Nipah virus outbreak occurred in India in 2001, resulting in 66 cases of encephalitis with a case-fatality of 74% [7]. Epidemiological data suggest that 75% of the Nipah virus patients in this outbreak were exposed to the virus within a hospital setting, with human-to-human transmission as the most likely route [7]. Since 2001, outbreaks of Nipah virus have occurred almost every year in Bangladesh. Clinical presentation of Nipah virus in Bangladesh is somewhat different from that in Malaysia, with a higher proportion of respiratory disease and a higher case-fatality rate of up to 90% [8]. No intermediate host was implicated in the Nipah virus outbreaks in India and Bangladesh. Rather, epidemiological data suggest transmission of Nipah virus from bats to humans through the consumption of fruit or date palm sap contaminated by infected fruit bats [9], [10]. In addition, human-to-human transmission occurred on a larger scale during outbreaks in India and Bangladesh compared to Malaysia. For the Nipah virus outbreaks in Bangladesh between 2001 and 2007, it was estimated that ∼50% of Nipah virus cases were the result of human-to-human transmission events [11]. Since such a large proportion of Nipah cases was likely the result of human-to-human transmission it is important to understand the mode of transmission of Nipah virus and implement measures to prevent it in future outbreaks. Nipah virus has been isolated from human urine, saliva, nasal and oropharyngeal secretions and epidemiological data suggest that direct contact with these secretions of Nipah virus spreaders resulted in greater risk of Nipah virus infection. Three potential modes of human-to-human transmission of Nipah virus could be transmission via fomites, direct contact or aerosols.

In this study we assessed the potential of human-to-human transmission of Nipah virus in the Syrian hamster model. The Syrian hamster has been shown to replicate both the respiratory and neurological symptoms seen in humans [12], [13]. Through systematic transmission studies in the Syrian hamster model, we show that direct contact is the most efficient route of Nipah virus transmission.

## Methods

### Ethics statement

All animal experiments were approved by the Institutional Animal Care and Use Committee of the Rocky Mountain Laboratories (ASP #2011-03), and performed following the guidelines of the Association for Assessment and Accreditation of Laboratory Animal Care, International (AAALAC) by certified staff in an AAALAC-approved facility.

Virus and cells. Nipah virus (strain Malaysia) was kindly provided by the Special Pathogens Branch of the Centers for Disease Control and Prevention, Atlanta, Georgia, United States and propagated in VeroE6 cells in DMEM (Sigma) supplemented with 10% fetal calf serum (Hyclone, Logan), 1 mM L-glutamine (Lonza), 50 U/ml penicillin and 50 µg/ml streptomycin (Gibco).

### Animal experiments

To study the relation between inoculation dose and Nipah virus shedding, 3 groups of 18 6–8 week old female Syrian hamsters (HsdHan^tm^:AURA, Harlan Laboratories) were inoculated intranasally with 10^3^, 10^5^ or 10^7^ TCID_50_ of Nipah virus in a total volume of 100 µl. On days 2 and 4 post inoculation 6 animals were euthanized and lungs, trachea and nasal turbinates were collected for virologic and histopathologic analysis. Daily nasal, oropharyngeal, urinogenital and rectal swabs were obtained from the remaining 6 hamsters. Swabs were collected in vials containing 1 ml DMEM supplemented with 50 U/ml penicillin and 50 µg/ml streptomycin. Hamsters were euthanized on day 14 post inoculation or earlier upon signs of severe infection.

For fomite transmission experiments, eight 6–8 week old female singly housed Syrian hamsters, housed in a plastic cage with wood shavings, a feeder and a water bottle, were inoculated intranasally with 10^7^ TCID_50_ in a total volume of 100 µl. Nasal and oropharyngeal swabs were obtained daily to monitor the infectious status. On day 4 post inoculation, hamsters were euthanized and a single naïve hamster was placed in each cage. Bodyweight of these hamsters was determined daily and nasal and oropharyngeal swabs were taken until swabs were PCR-negative on three consecutive days. Naïve hamsters were euthanized upon signs of severe disease or four weeks post exposure.

For direct contact transmission experiments, eight 6–8 week old female singly housed Syrian hamsters were inoculated intranasally with 10^7^ TCID_50_ in a total volume of 100 µl. On day 1 post inoculation, a naïve hamster was added to each cage. Nasal and oropharyngeal swabs were obtained from inoculated and naïve hamsters daily and bodyweight of naïve hamsters was determined. On signs of severe disease, inoculated and naïve hamsters were euthanized; remaining hamsters were euthanized four weeks post exposure.

For aerosol transmission experiments, eight 6–8 week old female Syrian hamsters were inoculated intranasally with 10^7^ TCID_50_ in a total volume of 100 µl and singly housed in specially designed aerosol transmission cages. On day 1 post inoculation, a naïve hamster was placed on the opposite side of the inoculated hamster. The hamsters were separated by two stainless steel grids, allowing airflow from the inoculated to the naive hamster but preventing direct contact and fomite transmission. Nasal and oropharyngeal swabs were obtained from inoculated and naïve hamsters daily and bodyweight of naïve hamsters was determined. On signs of severe disease, inoculated and naïve hamsters were euthanized; remaining hamsters were euthanized four weeks post exposure.

### Virus titrations

Virus titrations were performed by end-point titration in VeroE6 cells. VeroE6 cells were inoculated with tenfold serial dilutions of swab medium or tissue homogenates. One hour after inoculation, the inoculum was removed and replaced with 200 µl DMEM supplemented with 10% fetal calf serum (Hyclone, Logan), 1 mM L-glutamine (Lonza), 50 U/ml penicillin and 50 µg/ml streptomycin (Gibco). Three days after inoculation, cytopathic effect (CPE) was scored and the TCID_50_ was calculated from 5 replicates by the method of Spearman-Karber. Tissue homogenates were prepared by adding 1 ml DMEM to the weighed tissue and homogenizing using a TissueLyzer II (Qiagen). Homogenates were centrifuged to clear the homogenate before inoculating cells.

### Histopathology and immunohistochemistry

Histopathology and immunohistochemistry was performed on hamster tissues. Anaesthetized hamsters were euthanized by exsanguination. Necropsies and tissue sampling were performed according to a standard protocol approved by the Institutional Biosafety Committee. After fixation for 7 days in 10% neutral-buffered formalin and embedding in paraffin, tissue sections were stained with hematoxylin and eosin (H&E) staining and an immunohistochemical method using a rabbit polyclonal antiserum against the Nipah virus nucleoprotein [14] (1∶5000; kindly provided by L. Wang, CSIRO Livestock Industries, Australian Animal Health Laboratory, Australia) as a primary antibody for detection of Nipah virus antigen. For the histopathological analysis of the nasal turbinates (NT) whole hamster skulls were used. The skulls were decalcified using a 20% EDTA solution in sucrose (Newcomer Supply) and allowed to sit at room temperature for 3 weeks. The 20% EDTA/sucrose solution was changed ×2 prior to gross sectioning the skull. The following tissues were examined: NT, trachea and lungs. Lesions were assigned a subjective score from 0 to 4 based on the percentage of the tissue that was immunopositive. The slides were evaluated by a veterinary pathologist.

### Quantitative PCR

RNA was extracted from swab samples using the NucleoSpin 96 Virus Core kit (Macherey-Nagel) and a Corbett Robotics model CAS 1820 automatic RNA extractor. RNA was eluted in 100 µl. 5 µl RNA was used in a one-step real-time RT-PCR targeted at the NP gene using the Rotor-Gene™ probe kit (Qiagen) according to instructions of the manufacturer (primer sequences are available on request). In each run, standard dilutions of a titered virus stock were run in parallel, to calculate TCID_50_ equivalents in the samples.

### Virus neutralization assay

Two-fold serial dilutions of heat-inactivated hamster sera were prepared in DMEM containing 2% fetal calf serum, 1 mM L-glutamine, 50 U/ml penicillin and 50 µg/ml streptomycin and 100 TCID_50_ of Nipah virus was added. After 1 hr at 37°C, this mix was added toVeroE6 cells. Three days after inoculation, wells were scored for CPE. The virus neutralization titer was expressed as the reciprocal value of the highest dilution of the serum, which still inhibits Nipah virus replication.

### ELISA

Immuno-globulin G antibody responses were measured in an enzyme-linked immunosorbent assay (ELISA) using Nipah virus Malaysia. Nipah virus-containing cell culture supernatant was concentrated and purified by spinning two hours at 21000 rpm over a 20% sucrose cushion. The pellet was resuspended in PBS and triton X-100 was added to a final concentration of 1%. This suspension was then used to coat immuno 96 microwell maxisorp plates (NUNC) at 4°C overnight. Subsequently, plates were blocked with 5% skim milk in PBS containing 0.05% Tween 20 (PBST) for 1.5 hours at 4°C. After 3 washes with PBST, 50 µL of diluted serum samples were added, and the plates were incubated for 1 hour at 37°C. Bound antibodies were detected after 3 washes using an anti-hamster secondary antibody conjugated with horseradish peroxidase (HRP; KPL). Following incubation for 1 hour at 37°C, bound HRP was detected using the ABTS® Peroxidase Substrate System (KPL). The absorbance at 405 nm was measured using a microplate spectrophotometer. Sera were considered positive when absorbance was higher than three standard deviations above the mean of negative control sera.

## Results

### Shedding of Nipah virus in infected hamsters

**Figure 1:**
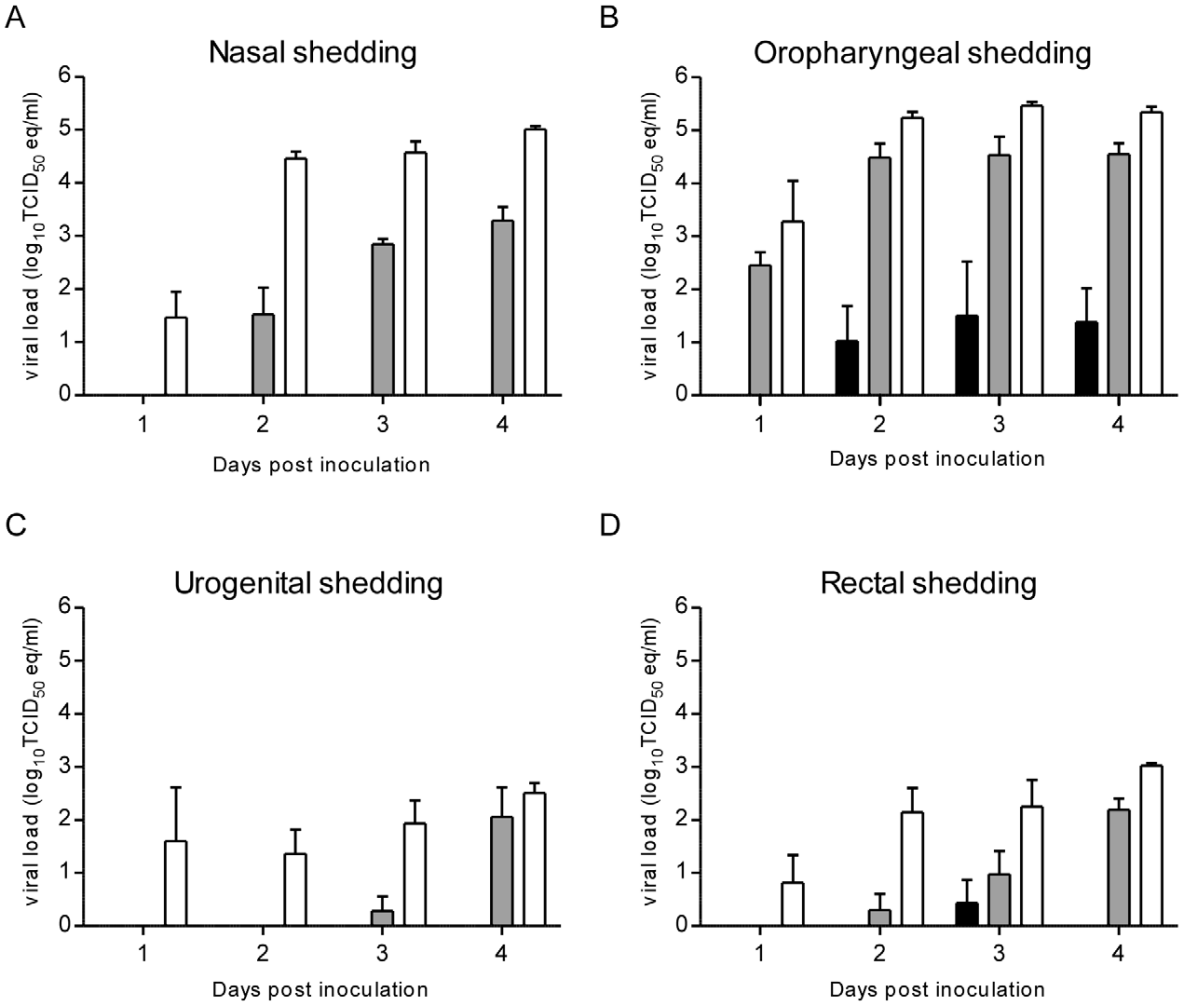
Shedding of Nipah virus RNA in inoculated hamsters. Groups of 6 hamsters were inoculated intranasally with 10^3^ (black bars), 10^5^ (grey bars) or 10^7^ TCID_50_ (white bars) Nipah virus. Nasal (A), oropharyngeal (B), urogenital (C) and rectal (D) swabs were collected daily and viral load in the swabs was determined as TCID_50_ equivalents by real-time RT-PCR. Geometric mean viral loads are displayed; error bars indicate standard deviation.

In order to determine the dose of Nipah virus to be used for transmission experiments, hamsters were inoculated intranasally with three different doses of the Malaysia strain of Nipah virus, 10^3^, 10^5^ or 10^7^ TCID_50_, to select the dose that resulted in the highest amount of Nipah virus shed from the nose, throat, urogenital tract and rectum. Upon intranasal inoculation of hamsters with 10^3^ TCID_50_, limited shedding of Nipah virus was observed in nasal, oropharyngeal, urogenital tract and rectal swabs in five out of six hamsters (Figures 1 and S1). One out of six hamsters did not shed any Nipah virus during the 14 days duration of the experiment. Two out of 6 hamsters showed weight loss in the days before presentation of neurological signs and were euthanized according to humane endpoint scoring criteria on days 8 and 14 post inoculation (Figure S2). The remaining three hamsters all shed virus on several days, although not all four swabs per time point were positive for all hamsters.

With a dose of 10^5^ TCID_50_ of Nipah virus, 5 out of 6 hamsters lost bodyweight; 2 hamsters survived until the end of the experiment on 14 dpi, the other four hamsters had to be euthanized due to severity of disease on 9 (three hamsters) and 12 dpi (Figure S2). In real-time RT-PCR, all six hamsters had positive nasal, oropharyngeal, urogenital and rectal swabs (Figures 1 and S1), starting at 2 dpi for the nasal shedding, 1 dpi for the oropharyngeal shedding, 3 dpi for the urogenital shedding and 2 dpi for the rectal shedding (Figure 1). Viral load in the oropharyngeal swabs was highest compared to the swabs obtained from the other orifices.

Upon inoculation with 10^7^ TCID_50_ of Nipah virus, hamsters had to be euthanized due to severity of disease on days 4 and 5 post inoculation (Figure S2). Virus shedding was observed starting at 1 dpi for all hamsters and all four swabs. Viral load in throat, nose, urogenital and rectal swabs were significantly higher for animals inoculated with 10^7^ TCID_50_ than from animals inoculated with either 10^3^ TCID_50_ or 10^5^ TCID_50_ of Nipah virus (2-way ANOVA, nose p<0.001, throat p<0.001, urogenital p<0.001 and rectal p<0.001, for both 10^7^ TCID_50_ vs 10^3^ TCID_50 vs_ and 10^7^ TCID_50_ vs 10^5^ TCID_50_).

**Figure 2:**
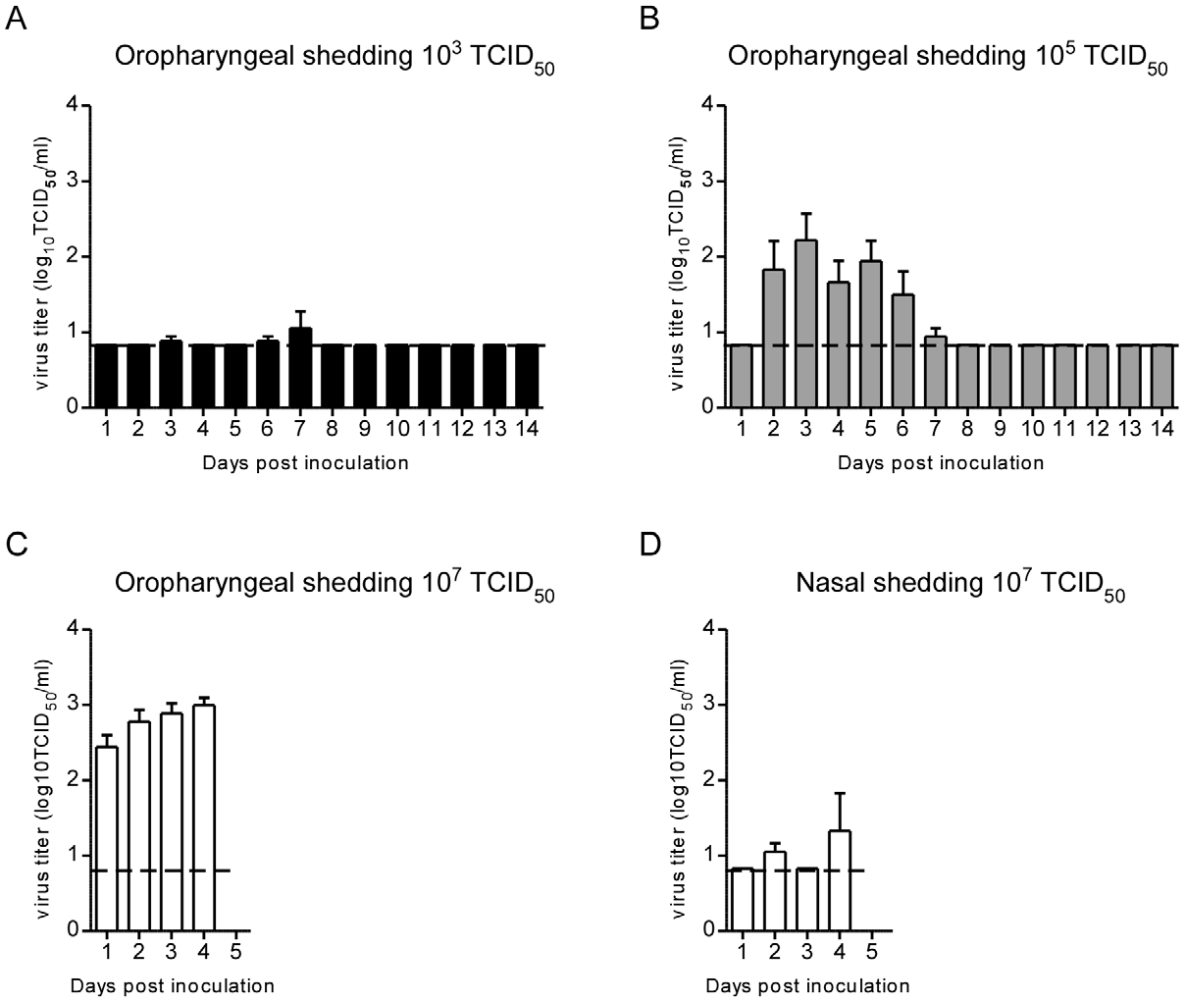
Shedding of Nipah virus from the respiratory tract. Virus titers in oropharyngeal swabs from hamsters inoculated with 10^3^ TCID_50_ (A); virus titers in oropharyngeal swabs from hamsters inoculated with 10^5^ TCID_50_ (B) and virus titers in oropharyngeal (C) and nasal (D) swabs from hamsters inoculated with 10^7^ TCID_50_. Nipah virus titers were determined on VeroE6 cells for real-time RT-PCR positive swabs by means of end-point titration. Geometric mean titers are displayed; error bars indicate standard deviation. To calculate the geometric mean, the cutoff value was used for negative swabs. The dotted line indicates cutoff value.

Virus titrations were performed on all PCR-positive swabs. For all infectious doses, urogenital and rectal swabs were negative upon virus titration. Upon inoculation with 10^3^ or 10^5^ TCID_50_ of Nipah virus, only oropharyngeal swabs were positive (Figure 2). With a dose of 10^3^ TCID_50_ only four swabs were positive, three of which in the animal that was euthanized on day 8. On day 2–5 post inoculation, oropharyngeal swabs of all six animals inoculated with 10^5^ TCID_50_ of Nipah virus were positive in virus titrations. With a dose of 10^7^ TCID_50_, Nipah virus could be detected in the nasal swabs of 5 out of 6 hamsters; oropharyngeal swabs of all hamsters were positive in virus isolation (Figure 2). Comparison of the total amounts of virus shed during the oropharyngeal shedding period indicated that a significantly higher amount of virus was shed with a dose of 10^7^ TCID_50_ compared to either of the two other doses in the first 4 dpi (area under curve analysis 0.2639 (95% confidence intervals of −0.2322 and 0.7600) for 10^3^ TCID_50_ vs. 4.958 (95% confidence intervals of 3.040 and 6.875) for 10^5^ TCID_50_ vs 7.44 (95% confidence intervals of 5.809 and 9.079) for 10^7^ TCID_50_, 1-way ANOVA p<0.0001).

**Figure 3:**
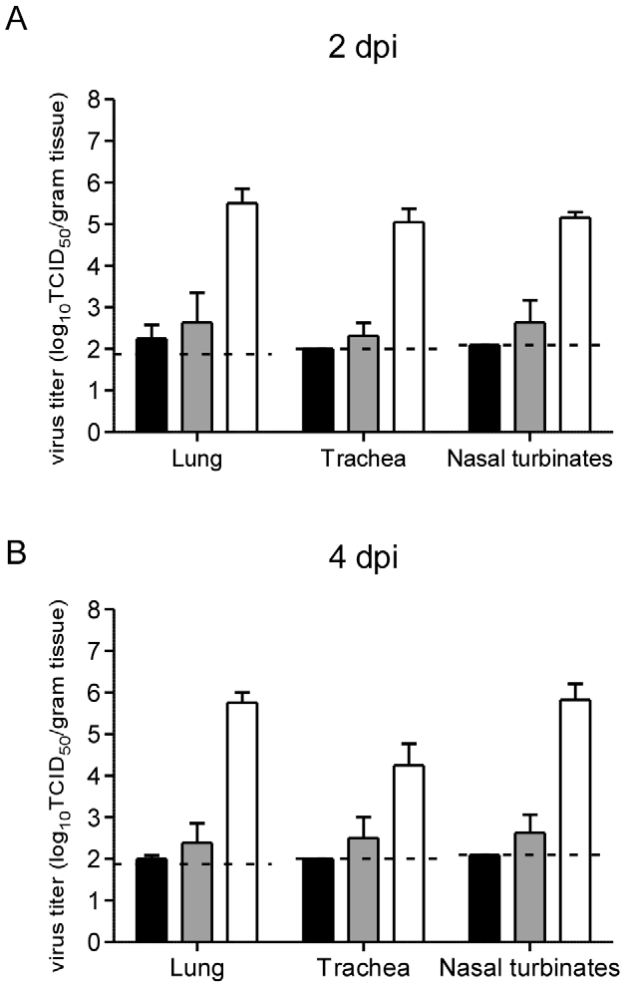
Nipah virus titers in respiratory tract tissues of inoculated hamsters. Virus titers in lung, trachea and nasal turbinates of hamsters inoculated intranasally with 10^3^ (black bars), 10^5^ (grey bars) or 10^7^ TCID_50_ (white bars) Nipah virus at 2 dpi (A) and 4 dpi (B). Nipah virus titers were determined on VeroE6 cells by means of end-point titration. Geometric mean titers are displayed; error bars indicate standard deviation. To calculate the geometric mean, the cutoff value was used for negative tissues. The dotted line indicates cutoff value.

On 2 and 4 dpi, 6 hamsters of each group were euthanized and virus titers in the nasal turbinates, trachea and lungs were determined for 3 of these animals; the remaining three hamsters were used for histopathological analyses. Only with the inoculum of 10^7^ TCID_50_ could Nipah virus be isolated in all three tissues of all three hamsters. The inoculum of 10^7^ TCID_50_ of Nipah virus showed significantly higher virus titers for 2 dpi and 4 dpi compared to the other 2 inoculum doses (2-way ANOVA, nasal turbinates 10^3^ TCID_50_ vs. 10^7^ TCID_50_, p<0.0001 and 10^5^ TCID_50_ vs. 10^7^ TCID_50_, p<0.0001, trachea 10^3^ TCID_50_ vs. 10^7^ TCID_50_, p<0.01 and 10^5^ TCID_50_ vs. 10^7^ TCID_50_, p<0.05 and lungs 10^3^ TCID_50_ vs. 10^7^ TCID_50_, p<0.0001 and 10^5^ TCID_50_ vs. 10^7^ TCID_50_, p<0.0001 (Figure 3)).

**Figure 4:**
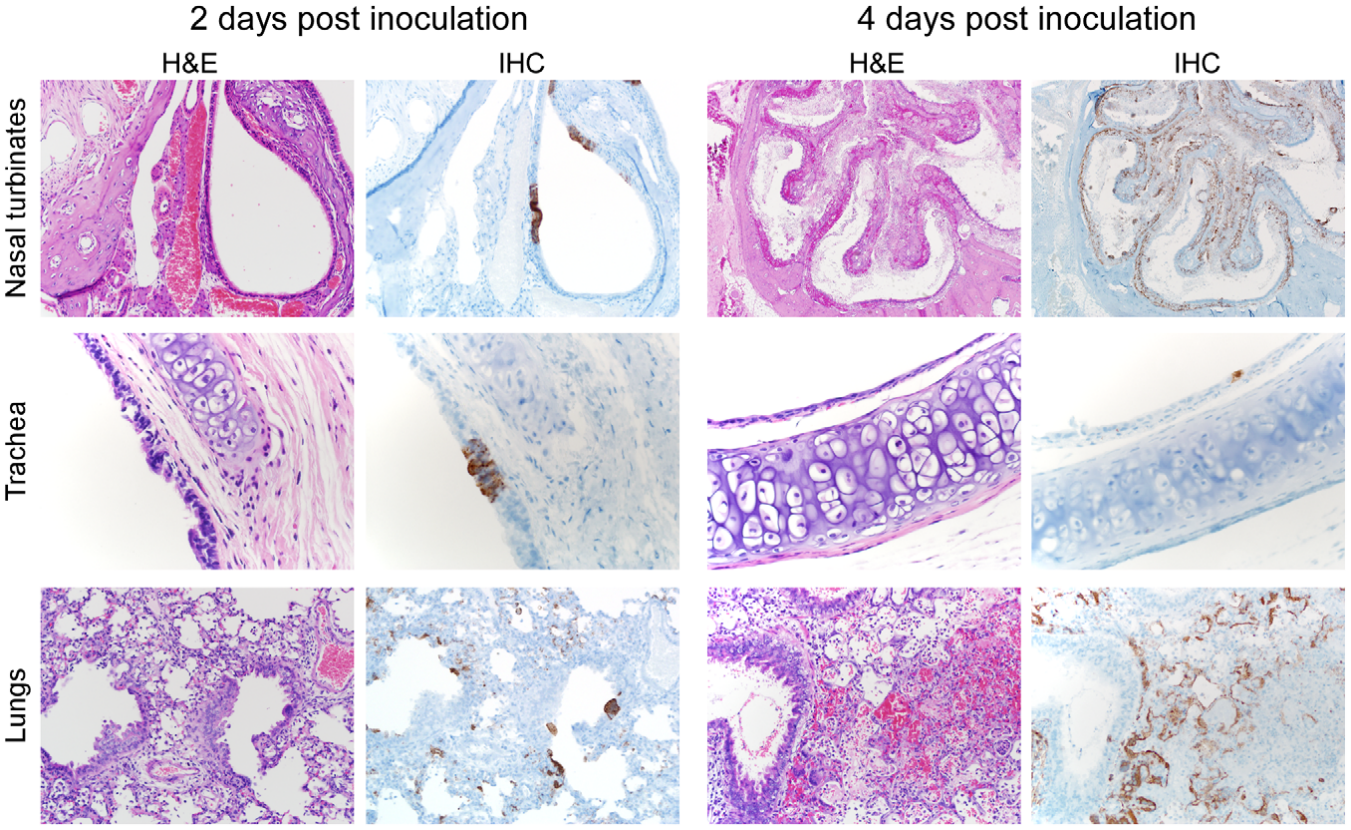
Immunohistochemical analysis of respiratory tract tissues of hamsters inoculated with Nipah virus. Hamsters were inoculated intranasally with 10^7^ TCID_50_ Nipah virus and respiratory samples were collected at 2 and 4 days post inoculation. Tissue sections of the nasal turbinates (A), trachea (B), and lung (C) were stained with a monoclonal antibody against Nipah virus nucleoprotein, which is visible as a red-brown staining.

In agreement with the absence of Nipah virus titers in the nasal turbinates, trachea and lungs of hamsters inoculated with 10^3^ TCID_50_ of Nipah virus did not show any pathological changes at 2 dpi (Table 1). One hamster showed a small number of alveolar epithelial cells and fewer macrophages in the lungs positive for Nipah viral antigen. At 4 dpi, one hamster showed moderate bronchointerstitial pneumonia characterized by multifocal bronchiolar epithelial degeneration and loss with thickening of adjacent alveolar septae by edema, fibrin and small numbers of neutrophils, macrophages and lymphocytes. One hamster inoculated with 10^5^ TCID_50_ of Nipah virus had a minimal bronchointerstitial pneumonia and associated viral antigen at 2 dpi and one animal had detectable viral antigen in the epithelium of the nasal turbinates (Table 1). At 4 dpi every hamster had acute rhinitis of both respiratory and olfactory epithelium, in two animals with associated viral antigen and one animal displayed mild multifocal bronchointerstitial pneumonia. The hamsters inoculated with 10^7^ TCID_50_ of Nipah virus demonstrated mild to moderate acute and necrotizing rhinitis in both respiratory and olfactory epithelium along with associated viral antigen at 2 dpi. All animals had multifocal chronic bronchointerstitial pneumonia with associated viral antigen. Similar pulmonary lesions were seen at day 4 dpi (Table 1, Figure 4). When comparing the different inoculum doses there was a clear dose effect with the higher dose inducing rhinitis and bronchointerstitial pneumonia at an earlier time frame when compared to the lower and intermediate doses (Table 1).

**Table 1:** Histopathology score based on immunohistochemistry of respiratory tissues of hamsters inoculated with Nipah virus.

	10^3^ TCID_50_						10^5^ TCID_50_						10^7^ TCID_50_				
	2 dpi			4 dpi			2 dpi			4 dpi			2 dpi			4 dpi	
Nasal turbinates	0	0	0	0	0	0	0	1	0	1	0	1	1	1	1	2	3
Trachea	0	0	0	0	0	1	0	0	0	0	0	1	0	1	1	1	0
Lungs	0	1	0	0	0	1	0	0	1	0	0	2	2	2	2	3	0

Hamsters were inoculated intranasally with 10^3^, 10^5^ or 10^7^ TCID50; three hamsters per dose per time point were sampled except for the 10^7^ TCID_5_0 group at 4 dpi, when only 2 animals remained; each column represents one animal.

Score: 0: no immunopositivity; 1: 1 to 25% of tissue immunopositive; 2: 26 to 50% of tissue immunopositive; 3: 51 to 75% of tissue immunopositive; 4: 76 to 100% of tissue immunopositive.

### Fomite transmission

**Figure 5:**
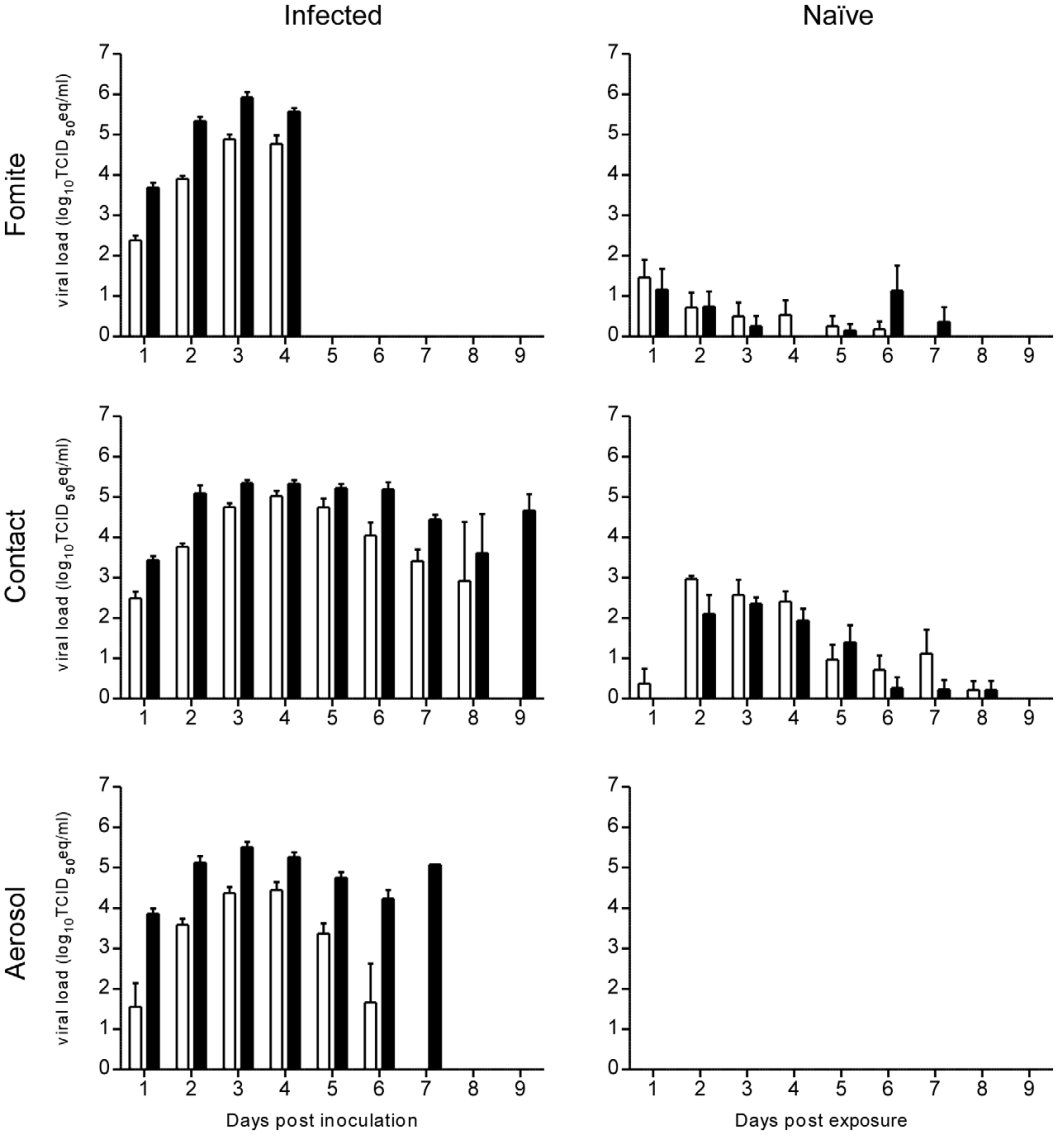
Transmission of Nipah virus. Shedding of Nipah virus in inoculated (left panels) and naïve (right panels) hamsters in fomite (A), direct contact (B) and aerosol (C) transmission setups. Nasal (white bars) and oropharyngeal (black bars) were collected daily; viral load in the swabs was determined as TCID_50_ equivalents by real-time RT-PCR. Geometric mean titers are displayed; error bars indicate standard deviation.

Based on the results of the studies described above, which showed significantly more Nipah virus shedding with the inoculum of 10^7^ TCID_50_, this dose was chosen for the following transmission experiments. To determine whether Nipah virus can be transmitted via fomites, eight singly housed hamsters were inoculated intranasally with 10^7^ TCID_50_ of Nipah virus. Nasal and oropharyngeal swabs were obtained daily. On 4 dpi, all inoculated hamsters were euthanized and a naïve hamster was placed in their cage, while leaving existing bedding, food and water bottles in place. Nasal and oropharyngeal swabs were obtained daily from the naïve hamsters. Swabs were analyzed by real time RT-PCR. Swabs from all eight naïve hamsters were positive on at least 2 days (Figure 5). The naïve hamsters did not show loss of bodyweight or other signs of disease; however, one hamster was found dead on day 7 post exposure. Histopathology of the respiratory tract and brain of this animal did not show any presence of Nipah virus, suggesting an unrelated cause of death. Remaining animals were euthanized four weeks post exposure. In a virus neutralization assay performed on the sera from these animals, no neutralizing antibodies against Nipah virus were detected; nor did the sera test positive in an ELISA using whole virus (data not shown).

### Direct contact transmission

To determine whether Nipah virus can be transmitted through direct contact, 8 singly housed hamsters were inoculated intranasally with 10^7^ TCID_50_ of Nipah virus. One day post inoculation, a naïve hamster was added to each cage. The naive hamsters did not lose bodyweight during the experiment. One hamster showed signs of disease on 10 dpi and was euthanized. The cause of death was not histologically apparent in this animal and was thus likely unrelated to Nipah virus infection. Nasal and oropharyngeal swabs were obtained from all hamsters. Swabs from all hamsters were positive on at least two days (Figure 5). More swabs of the direct contact hamsters were positive than of the fomite hamsters (35 vs. 14 for nasal swabs and 29 vs. 13 for oropharyngeal swabs). Remaining animals were euthanized four weeks post exposure. Upon histological examination, two hamsters showed minimal acute interstitial pneumonia characterized by small, nodular aggregates of macrophages and viable neutrophils that filled alveoli and mildly thickened adjacent alveolar septa (data not shown). In a virus neutralization assay performed on the sera from all eight hamsters, neutralizing antibodies were detected in three hamsters, with neutralizing titers ranging from 64–256. These sera also contained high titer antibodies as determined by ELISA, the remaining sera from hamsters in this group were negative.

### Aerosol transmission

To determine whether Nipah virus can be transmitted through aerosols, eight singly housed hamsters were inoculated intranasally with 10^7^ TCID_50_ of Nipah virus and kept in cages with a divider. One day post inoculation, a naïve hamster was added to the opposite side of the divider in each cage. The divider was specifically designed to allow airflow from the infected to the naïve hamster, but prevent contact and fomite transmission. Hamsters were swabbed daily. Between 4 and 7 dpi, all inoculated animals were euthanized due to respiratory distress. Nasal and oropharyngeal swabs of the naïve hamsters remained negative (Figure 5). Naïve hamsters were euthanized four weeks post exposure. In a virus neutralization assay performed on the sera from these animals, no neutralizing antibodies against Nipah virus were detected; nor did the sera test positive in an ELISA using whole virus (data not shown).

## Discussion

Since its' first emergence in 1999, outbreaks of Nipah virus have occurred almost every year. Introduction of Nipah virus into the human population and subsequent transmission within the human population appears to occur via multiple routes of introduction and transmission. Whereas the Malaysian Nipah virus outbreak was caused by an introduction of Nipah virus into the susceptible swine population and was subsequently transmitted to humans [6], the multiple outbreaks in India and Bangladesh appear to have been caused by direct transmission of Nipah virus from the natural reservoir without an amplifying intermediate host [9]. Based on epidemiological data, it has been suggested that both swine-to-human and human-to-human transmission of Nipah virus have played a major role in past Nipah virus outbreaks [1], [6], [7], [11], [15], [16], [17], [18], [19], [20], [21], [22].

Currently, most data on the human-to-human transmission of Nipah virus originate from investigations into the multiple outbreaks in Bangladesh, where human-to-human transmission has occurred frequently [11], [15], [16], [17], [18], [20], [22]. In the present study, we have gathered scientific data that strengthen these epidemiological observations. In a hamster model, efficient Nipah virus transmission was observed via direct contact between inoculated and naïve hamsters. The transmission was confirmed by the presence of neutralizing antibodies in the naïve hamsters. Although viral RNA was detected in nose and throat swabs obtained from naïve hamsters during the fomite transmission experiment, no virus replication or neutralizing antibodies were detected, suggesting that although there is a potential for fomite transmission it seemed very inefficient under the experimental conditions of our model. Within our model, no aerosol transmission of Nipah virus occurred as indicated by a lack of apparent signs of disease, virus shedding, neutralizing antibodies and presence of viral antigen in organs of exposed naïve hamsters.

In a previous study by Wong et al., transmission of Nipah virus strain Malaysia was not observed in a hamster model upon intraperitoneal (i.p.) inoculation with a dose of 10^5^ TCID_50_ [13]. Virus shedding in inoculated or naïve transmission hamsters was not tested and the absence of transmission was concluded based on the absence of disease signs and seroconversion in the naïve hamsters. This could either indicate that i.p. inoculation did not result in sufficient shedding of viable virus particles to allow transmission, since the virus would have to migrate to the nasal cavity or urinary bladder, that transmission efficiency is dose dependent, or a combination thereof.

Our data suggest that hamsters inoculated with 10^7^ TCID_50_ not only shed significantly more Nipah virus particles as determined by realtime RT-PCR than hamsters inoculated with lower doses, but more importantly also shed more viable Nipah virus as determined by virus titration. With the lower inoculum doses limited shedding of viable Nipah virus was observed, indicating that the inoculum dose may very well affect the ability of Nipah virus to transmit efficiently, through an effect on virus shedding. Our results indicate the importance of nasal and oropharyngeal shedding and transmission and are in line with previous experimental Nipah virus infections in pigs where virus excretion was also observed in inoculated and contact pigs, although the mechanism by which the transmission occurred was not investigated [23]. This suggests that the mode of transmission from pig-to-pig, pig-to-human and human-to-human are the same and is facilitated by direct contact with Nipah virus containing nasal and oropharyngeal secretions.

Interestingly, epidemiological data gathered during the Nipah virus outbreak in Malaysia have not identified human-to-human transmission in this outbreak, although four potential cases of nosocomial transmission have been reported [24], [25]. Our results show that Nipah virus strain Malaysia has the ability to transmit upon contact with nasal or oropharyngeal secretions during close social interactions. This is in agreement with the epidemiological data suggesting that the introduction of Nipah virus in the human population in Malaysia occurred upon direct contact with infected swine. The absence of disease signs in hamsters that were infected with Nipah virus via the direct contact route could be due to the relatively low inoculum dose as indicated by the low infectious virus titers shed by infected hamsters (Figure 2). As previously shown, a low inoculum dose can result in slow progression towards neurological disease after a limited transient respiratory infection [12], suggesting that the contact hamsters may have been euthanized before they presented with neurological disease signs. This may explain the epidemiology of the Malaysian Nipah virus outbreak, in which very limited human-to-human transmission was observed, where patients may have experienced little respiratory involvement and thus likely did not shed amounts of virus sufficient for human-to-human transmission. The absence of human-to-human transmission in the Malaysia outbreak as compared to more prominent human-to-human transmission in Bangladesh could potentially be caused by an intrinsic difference in transmissibility of the respective virus [26], e.g. the ability of the virus to efficiently replicate in humans and be shed with higher titers could potentially facilitate more efficient human-to-human transmission. Alternatively, different cultural or health care practises may underlie the observed difference in human-to-human transmission of Nipah virus strain Bangladesh and Nipah virus strain Malaysia. Experiments to compare the transmission efficiency of Nipah virus Bangladesh, which was not available to us, with Nipah virus Malaysia should be performed to gain insight in the cause of differences in observed human-to-human transmission of Nipah virus isolated from Malaysia vs. Bangladesh.

Evidence for vertical transmission was observed in a cat model of Nipah virus disease. In a pregnant cat, infectious virus was detected in placental tissue and Nipah virus genomic RNA was detected in fetal tissue [27]. Since Hendra virus has been isolated from fetal material and uterine fluid of Pteropus bats, vertical transmission may be an important transmission route in the natural reservoir of Nipah and Hendra virus. It has been suggested that Nipah virus-infected fetal tissues or fluid may play a role in the zoonotic transmission of Nipah virus from bats to other mammals [28], [29]. Although vertical transmission of Nipah virus in bats or incidental hosts of Nipah virus may occur, it seems unlikely that vertical transmission would play an important role in the perpetuation of Nipah virus outbreaks in humans.

The experimental data on the route of transmission of Nipah virus presented here have important public health implications with regards to the risk assessment and management of future Nipah virus outbreaks. In addition, this novel transmission model can be used to evaluate the efficacy of outbreak intervention strategies, such as vaccination and antiviral therapies. Whereas current intervention strategies are predominantly focused at post exposure treatment, the ability to efficiently block transmission and thereby spread of the outbreak is currently not assessed in antiviral or vaccination treatment strategies [14], [30], [31], [32], [33]. The novel contact transmission model not only allows an experimental approach to understanding the biotic and abiotic factors underlying human-to-human transmission, but in addition might allow incorporation of the role of foodborne transmission of Nipah virus Bangladesh through contaminated date palm juice, that is suggested to play a major role in the introduction of Nipah virus into the human population in Bangladesh [9], [10]. Moreover, the hamster transmission model can be used to test the effect of intervention strategies on the containment of the outbreak by preventing transmission. As such, the hamster transmission model will not only contribute to the basic understanding of Nipah transmission, it will also be of value from a public health perspective.

## Supporting Information

Figure S1 **Shedding of Nipah virus RNA in inoculated hamsters.** Groups of 6 hamsters were inoculated intranasally with 10^3^ (black bars), 10^5^ (grey bars) or 10^7^ TCID_50_ (white bars) Nipah virus. Nasal (A), oropharyngeal (B), urogenital (C) and rectal (D) swabs were collected daily for 14 days and viral load in the swabs was determined as TCID_50_ equivalents by real-time RT-PCR. Geometric mean viral loads are displayed; error bars indicate standard deviation.
(TIF)

Figure S2 **Loss of bodyweight and survival in hamsters inoculated with Nipah virus.** Loss of bodyweight (A) and survival (B) after intranasal inoculation of with 10^3^ (solid line, circles), 10^5^ (dashed line, squares) or 10^7^ (small dashed line, triangles) TCID50 Nipah virus are plotted. Hamsters were weighed daily, and the percentage of body weight was calculated relative to the weight at time of inoculation. The percentage of mice surviving the infection is shown as a function of time.
(TIF)
