# Supplementary figures and images for: Nipah Virus Transmission in a Hamster Model
Source: PLoS Negl Trop Dis. 2011 Dec 13;5(12):e1432. doi: 10.1371/journal.pntd.0001432 (PMC3236726; doi:10.1371/journal.pntd.0001432)

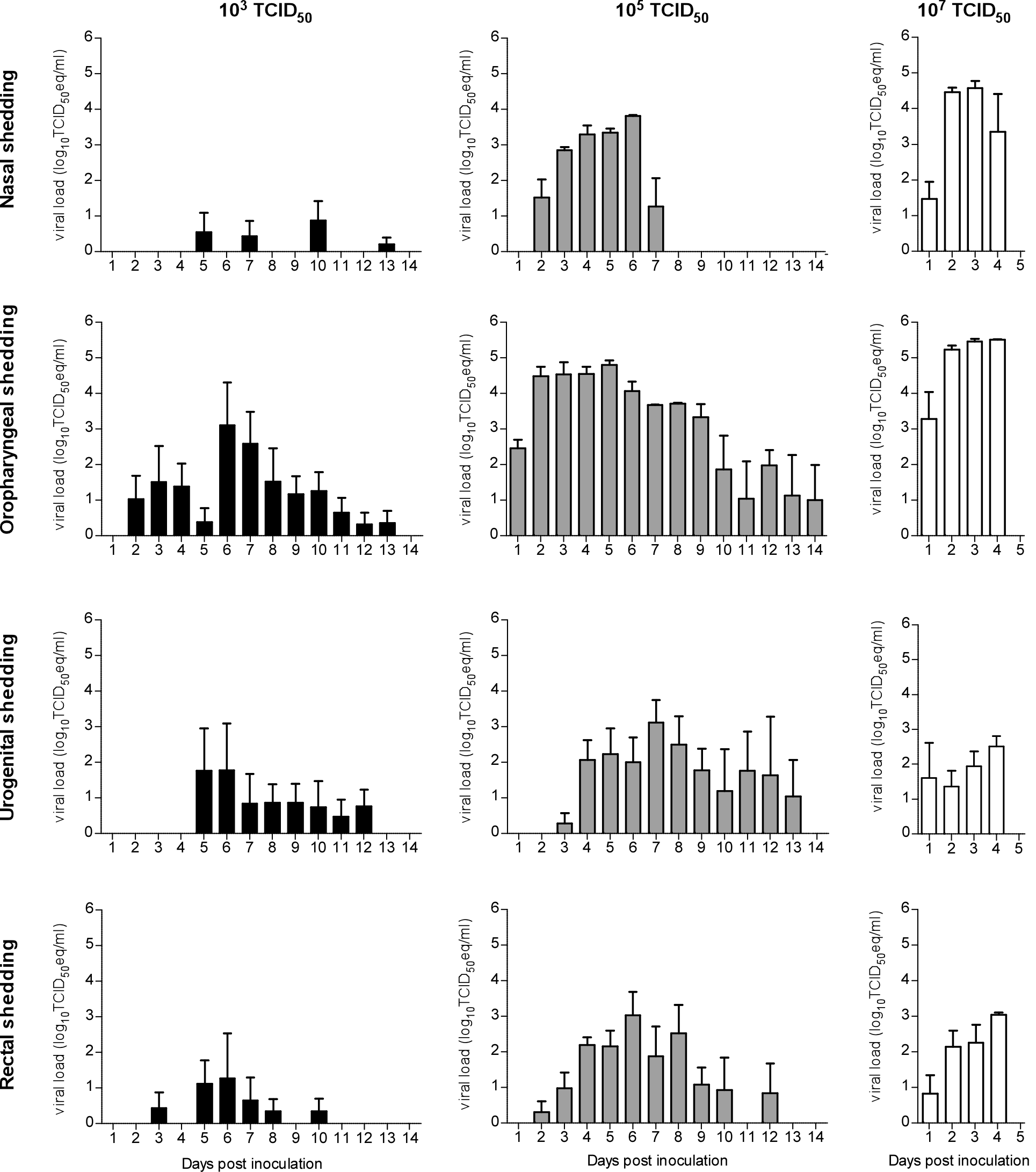

Supplement: Figure S1 — Shedding of Nipah virus RNA in inoculated hamsters. Groups of 6 hamsters were inoculated intranasally with 103 (black bars), 105 (grey bars) or 107 TCID50 (white bars) Nipah virus. Nasal (A), oropharyngeal (B), urogenital (C) and rectal (D) swabs were collected daily for 14 days and viral load in the swabs was determined as TCID50 equivalents by real-time RT-PCR. Geometric mean viral loads are displayed; error bars indicate standard deviation. (TIF) [file pntd.0001432.s001.tif]

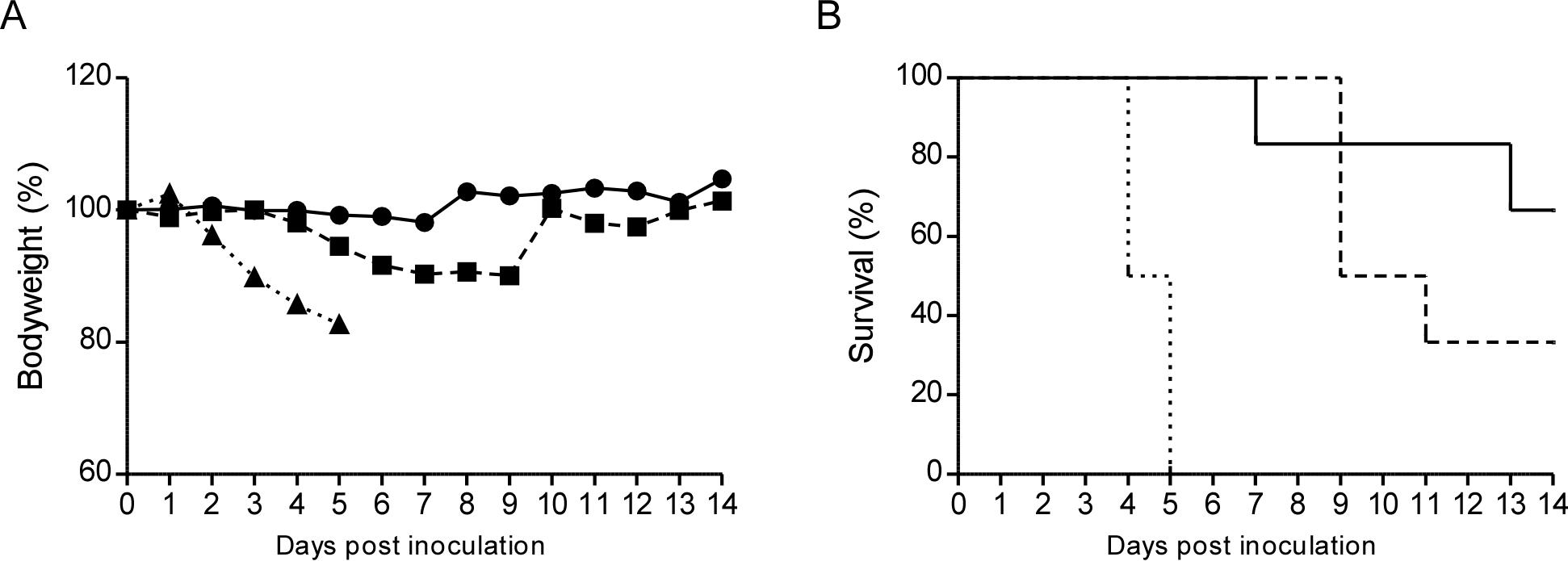

Supplement: Figure S2 — Loss of bodyweight and survival in hamsters inoculated with Nipah virus. Loss of bodyweight (A) and survival (B) after intranasal inoculation of with 103 (solid line, circles), 105 (dashed line, squares) or 107 (small dashed line, triangles) TCID50 Nipah virus are plotted. Hamsters were weighed daily, and the percentage of body weight was calculated relative to the weight at time of inoculation. The percentage of mice surviving the infection is shown as a function of time. (TIF) [file pntd.0001432.s002.tif]
